# Respiratory support in patients with acute respiratory distress syndrome: an expert opinion

**DOI:** 10.1186/s13054-017-1820-0

**Published:** 2017-09-12

**Authors:** Davide Chiumello, Laurent Brochard, John J. Marini, Arthur S. Slutsky, Jordi Mancebo, V. Marco Ranieri, B. Taylor Thompson, Laurent Papazian, Marcus J. Schultz, Marcelo Amato, Luciano Gattinoni, Alain Mercat, Antonio Pesenti, Daniel Talmor, Jean-Louis Vincent

**Affiliations:** 1 Dipartimento di Scienze della Salute, Università degli Studi di Milano, Milan, Italy; 2 Keenan Research Centre, Li Ka Shing Knowledge Institute, St Michael’s Hospital, Toronto, ON Canada; 3 Interdepartmental Division of Critical Care Medicine, University of Toronto, Toronto, ON Canada; 4 University of Minnesota, Minneapolis, Saint Paul, MN USA; 5 University of Montreal and Department of Intensive Care, Centre Hospitalier Université de Montréal, Montréal, QC Canada; 6 Department of Anesthesia and Critical Care Medicine, Sapienza University of Rome, Policlinico Umberto I Hospital, Viale del Policlinico 155, 00161 Rome, Italy; 7 Division of Pulmonary and Critical Care Medicine, Massachusetts General Hospital, Boston, MA USA; 8 Réanimation des Détresses Respiratoires et Infections Sévères, Hôpital Nord—Assistance Publique—Hôpitaux de Marseille Aix-Marseille Université, Marseille, France; 9 Mahidol Oxford Research Unit (MORU), Faculty of Tropical Medicine, Mahidol University, Bangkok, Thailand; 10 Laboratório de Pneumologia LIM-09, Disciplina de Pneumologia, Heart Institute (InCor) Hospital das Clínicas da Faculdade de Medicina da Universidade de São Paulo, São Paulo, Brazil; 11 Department of Anesthesiology, Emergency and Intensive Care Medicine, University of Göttingen, Robert-Koch-Straße 40, 37075 Göttingen, Germany; 12 CHU d’Angers, Réanimation Médicale et Médecine Hyperbare, Angers, France; 13 Department of Anesthesia, Critical Care and Emergency, Fondazione IRCCS Ca’ Granda Ospedale Maggiore Policlinico, Via F. Sforza 35, 20122 Milan, Italy; 14 Department of Pathophysiology and Transplantation, University of Milan, Milan, Italy; 15 Department of Anesthesia and Critical Care Medicine, Beth Israel Deaconess Medical Center, Boston, MA USA; 16 Department of Intensive Care, Erasme Hospital, Université libre de Bruxelles, Route de Lennik 808, 1070 Brussels, Belgium

**Keywords:** Ventilator support, Ventilator-induced lung injury, Tidal volume, Positive end-expiratory pressure, Esophageal pressure, Extracorporeal membrane oxygenation, Weaning

## Abstract

Acute respiratory distress syndrome (ARDS) is a common condition in intensive care unit patients and remains a major concern, with mortality rates of around 30–45% and considerable long-term morbidity. Respiratory support in these patients must be optimized to ensure adequate gas exchange while minimizing the risks of ventilator-induced lung injury. The aim of this expert opinion document is to review the available clinical evidence related to ventilator support and adjuvant therapies in order to provide evidence-based and experience-based clinical recommendations for the management of patients with ARDS.

## Background

Since its first description in 1967 [1], acute respiratory distress syndrome (ARDS) has been redefined several times. According to the latest consensus (Berlin Definition), ARDS is defined as the presence within 1 week of a known clinical insult, of acute arterial hypoxemia (PaO_2_/FiO_2_ ≤ 300 mmHg) with a minimum requirement of 5 cmH_2_O positive end-expiratory pressure (PEEP), plus the presence of bilateral radiographic opacities not entirely explained by cardiac failure or fluid overload [2]. ARDS is classified as mild (200 < PaO_2_/FiO_2_ ≤ 300 mmHg), moderate (100 < PaO_2_/FiO_2_ ≤ 200 mmHg) or severe (PaO_2_/FiO_2_ ≤ 100 mmHg). Approximately 25% of mechanically ventilated intensive care unit (ICU) patients have ARDS [3] and, despite advances in supportive care, ICU mortality rates are still 35–40% and increase with the severity of hypoxemia. Many patients with ARDS also have persistent morbidity after discharge [4].

To minimize the risks of ventilator-induced lung injury (VILI) in these patients [5] and thus optimize outcomes, several interventions have been proposed, including use of low tidal volume ventilation [6], application of sufficient PEEP [7] and, in severe cases, prone positioning [8], neuromuscular blocking agents [9] and extracorporeal membrane oxygenation (ECMO) [10]. However, even simple low tidal volume ventilation is not always applied [3].

**Fig. 1:**
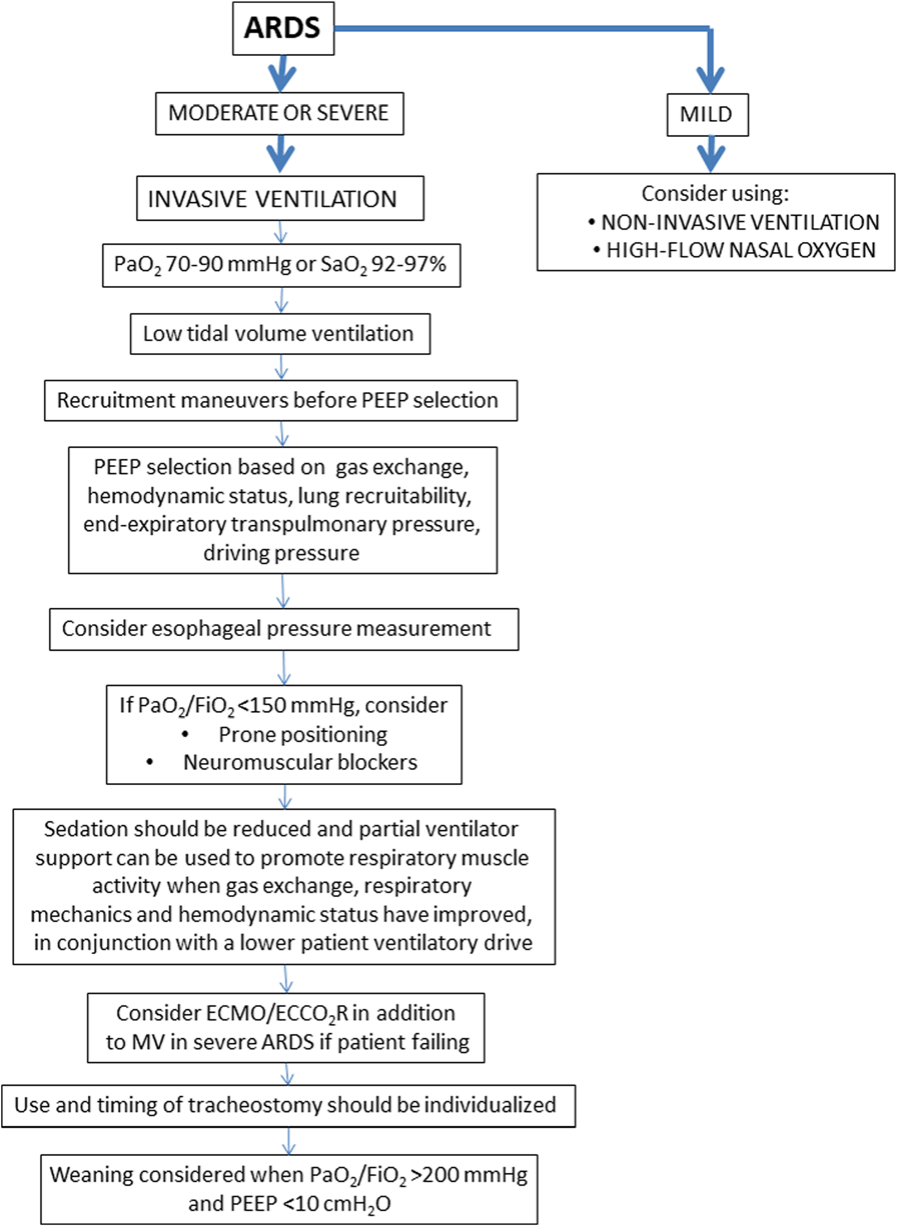
Suggested ventilator support options and adjuvant therapies in patients with acute respiratory distress syndrome (ARDS). ECCO_2_R extracorporeal carbon dioxide removal, ECMO extracorporeal membrane oxygenation, FiO_2_ inspired oxygen fraction, PEEP positive end-expiratory pressure, MV mechancial ventilation, PaO_2_ arterial partial pressure of oxygen, SaO_2_ arterial oxygen saturation

Recent ARDS guidelines have made some recommendations regarding ventilator management, without always reaching a consensus between members of the panel [11]. In this expert opinion document, we review the available clinical evidence related to ventilator support and adjuvant therapies in order to provide evidence-based and experience-based clinical recommendations for the management of patients with ARDS (Fig. 1).

## Statements

### Noninvasive support, with close monitoring, is a reasonable initial approach in less severely ill patients with ARDS

#### Rationale and literature findings

In a randomized trial of adult patients admitted to the ICU for acute hypoxemic, nonhypercapnic respiratory insufficiency, continuous positive airway pressure (CPAP) delivered by face mask was associated with an early improvement in oxygenation; however, it was not associated with a reduced need for intubation or with improved outcomes [12]. Indeed, noninvasive ventilation (NIV) can fail because of the severity of the disease, patient noncompliance or technical problems, particularly at the interface. To improve NIV success rates, the helmet has been proposed as an alternative interface compared to the face mask. In a multicenter, randomized controlled trial (RCT) conducted in four Italian centers in patients with severe hypoxemic acute respiratory failure due to pneumonia, helmet CPAP reduced the risk of meeting endotracheal intubation criteria compared to oxygen therapy but with no difference in outcome [13]. To explore the issue of the interface, a recent single-center RCT studied the effect of NIV delivered by helmet or face-mask among patients with ARDS. The authors concluded that helmet NIV was associated with significant reductions in intubation rates and 90-day mortality [14].

A recent trial compared high-flow nasal cannula (HFNC) oxygen, standard oxygen via a face mask and face-mask NIV in 310 patients with acute hypoxemic respiratory failure. The intubation rate was significantly lower with HFNC oxygen than with standard oxygen or NIV among patients with PaO_2_/FiO_2_ ≤ 200 mmHg at enrollment and, for the whole group (patients with PaO2/FiO2 ≤ 300 mmHg), patients managed with HFNC had improved survival. There were no differences in outcomes between NIV and standard oxygen [15].

HFNC can generate low levels of PEEP in the upper airways, decrease work of breathing and reduce dead space [16, 17]. It is an attractive technique as a first-line therapy to avoid intubation but the results need confirmation. In moderate ARDS, noninvasive support may be considered in selected cases; for example, in cognizant younger patients, in patients with a Simplified Acute Physiology Score (SAPS II) < 34 and in patients with ARDS not caused by pneumonia [18].

In all cases in which noninvasive support is used, patients should be monitored closely, as deterioration can occur abruptly [18]. Positive responses are usually evident soon after initiation. If there is no substantial improvement in gas exchange and respiratory rate within a few hours, invasive mechanical ventilation should be started without delay. Failure to recognize a lack of improvement during noninvasive support may result in further respiratory deterioration and/or cardiac arrest, often with devastating consequences. Moreover, noninvasive support in patients with a high respiratory drive may encourage excessive transpulmonary pressure swings, increasing the risk of patient-self-inflicted lung injury. A rapid shallow breathing index (RSBI) > 105 breaths/min/L may be associated with need for intubation in patients receiving NIV [19]. Monitored tidal volumes persistently > 9.5 ml/kg predicted body weight (PBW) suggest the need for intubation [20]. Delayed intubation is associated with increased mortality in patients with acute respiratory failure [21], but premature intubation in patients in whom noninvasive respiratory support is adequate exposes the patient to potentially unnecessary risks associated with invasive mechanical ventilation.

### PaO_2_ should be maintained within a normal range (e.g., between 70 and 90 mmHg) or SaO_2_ between 92 and 97%

#### Rationale and literature findings

Hypoxemia and hyperoxia can both be deleterious but clinicians tend to be more tolerant of hyperoxia. Hypoxemia represents a cardiovascular and hemodynamic stress and may limit oxygen delivery to the tissues, except in patients who are accustomed to hypoxemia as a result of chronic disease or residence at high altitude [22]. Long-term deleterious effects of hypoxemia, such as neuropsychological impairment, have also been suggested [23], but confirmatory research is needed. Hyperoxia may increase lung inflammation, can adversely affect the microcirculation and is associated with increased mortality rates in certain categories of patients [24, 25]. One certainty is that there is no known benefit to be achieved from hyperoxia and clinicians should target saturation values in the normal range.

### Low tidal volume ventilation, about 6 ml/kg based on predicted body weight, along with an airway plateau pressure ≤ 30 cmH_2_O should be targeted in most patients with ARDS

#### Rationale and literature findings

Although high tidal volumes (>10 ml/kg) and elevated airway plateau pressures (Pplat) may increase the amount of recruited volume at end expiration [26, 27], large tidal volume ventilation can result in overdistension and excessive lung stress, especially in ARDS patients [28]. In a seminal prospective RCT by the ARDS Network, a ventilatory strategy targeting a tidal volume of 6 ml/kg PBW and Pplat ≤ 30 cmH_2_O was associated with reduced mortality in patients with ARDS compared with a strategy targeting a tidal volume of 12 ml/kg PBW and Pplat ≤ 50 cmH_2_O [6]. Mechanical ventilation with excessive tidal volumes can induce a systemic and pulmonary inflammatory cytokine response that may be attenuated by a lung-protective strategy [29]. However, although setting the tidal volume according to PBW is an easy way to initiate protective ventilation, this strategy can result in different levels of lung stress and strain according to the amount and distribution of aerated lung tissue [30].

Some studies have suggested that tidal volumes even less than 6 ml/kg may be preferable [31], but higher PEEP levels may then be necessary to maintain oxygenation [32]. In one study, the combination of lower tidal volume and higher PEEP significantly reduced hospital mortality compared to higher tidal volume and lower PEEP [33]. The coexistence of severe acidosis may prevent strict adherence to these objectives unless addressed by other measures, such as the concurrent use of extracorporeal life support. Large increases in chest wall stiffness may cause Pplat to exceed the recommended upper limit of 30 cmH_2_O, even when lung stretch is not excessive.

Unfortunately, the evidence supporting lower tidal volumes is not always applied, with a recent large international survey showing that tidal volume was kept at < 7 ml/kg PBW in only about 50% of patients with ARDS [3]. It has been suggested that tidal volume should be titrated according to the PBW and not to the ideal body weight (IBW) because of a better relationship, in healthy subjects, of PBW with lung size [34]. However, in patients with ARDS, the proportion of the lung available for ventilation is markedly decreased, which is reflected by low respiratory-system compliance [35]. Therefore, it was recently suggested that tidal volume should be scaled to compliance using the driving pressure (∆*P* = Pplat – PEEP). Indeed driving pressure is the ratio of tidal volume to compliance, the latter indicating the “functional” size of the lung. Driving pressure predicts outcomes better than any other ventilatory parameters in patients with ARDS, with values exceeding 15 cmH_2_O of particular concern [36]. Thus, observation of a low driving pressure may reinforce the relaxation of strict tidal volume or Pplat targets in patients with conflicting clinical priorities (e.g., a patient with severe acidosis and high PEEP requirements).

### Recruitment maneuvers can be applied before PEEP selection or in case of abrupt derecruitment

#### Rationale and literature findings

Alveolar collapse is mainly generated by inflammatory lung edema, impairment of chest wall movement and surfactant deficiency. To recruit lung alveoli, one can apply a transient increase in inspiratory airway pressure to 40–45 cmH_2_O. Such recruitment maneuvers are an integral part of decremental selection of PEEP. Different types of recruitment maneuver, such as sustained inflation, intermittent sighs and stepwise increase in inspiratory pressure, have been suggested [37]; however, the optimal procedure and precise role of recruitment maneuvers has not yet been defined. In the majority of patients, a recruitment maneuver can improve oxygenation for a brief period of time without major side effects; however, routine application of recruitment maneuvers is not associated with a reduction in hospital mortality [38]. Some reports have shown limited effects of recruitment maneuvers when baseline PEEP levels exceed 10–12 cmH_2_O [39], but others have shown consistent effects even for baseline PEEP levels of around 17 cmH_2_O [40, 41]. For severely hypoxemic patients with evidence of recruitability following a recruitment maneuver, higher PEEP levels are probably required to maintain the benefit.

### PEEP selection should be based on various factors, including gas exchange, hemodynamics, lung recruitability, end-expiratory transpulmonary pressure and driving pressure

#### Rationale and literature findings

Use of PEEP usually improves gas exchange and helps reduce the need for high FiO_2_. In addition, appropriate levels may limit VILI, by maintaining lung recruitment, improving lung homogeneity [42] and reducing so-called atelectrauma attributed to repeated opening and closing of alveoli [43]. When applied with a constant tidal volume, PEEP simultaneously reduces the number of lung units exposed to stress but increases the stresses on those already open and on those which lie at the interface of closed and open tissue [44]. When applied with a constant Pplat, PEEP reduces the driving pressure and keeps the lung recruited. A meta-analysis showed that mortality was reduced when higher PEEP levels were applied in moderate and severe ARDS (PaO_2_/FiO_2_ ≤ 200 mmHg) [7].

PEEP selection criteria may include lung recruitability [45], end-expiratory transpulmonary pressure [46], respiratory system compliance and driving pressure [36]. Because the individual response to PEEP is highly variable [47], a test of two or three PEEP levels 15 min apart, without concomitant changes in oxygenation fraction or hemodynamic treatment, can help select optimal PEEP levels for individual patients; these tests should only be performed once the patient is stabilized. Physiological and clinical studies have suggested that decremental PEEP trials, preceded or not by recruitment maneuvers, commonly improve the physiological effects of PEEP (if compared to equivalent levels tested incrementally), thus also helping to disclose its effects on collapse prevention [45, 48]. Based on the available data, all PEEP values represent a compromise between the extent of recruitment and overdistension. Ongoing studies will help delineate the role of esophageal manometry and computations of end-expiratory transpulmonary pressure in guiding PEEP settings.

### Measurement of esophageal pressure should be considered during both controlled and assisted mechanical ventilation

#### Rationale and literature findings

The measurement of esophageal pressure, as a surrogate for pleural pressure, enables estimation of transpulmonary pressure (i.e., the distending pressure across the lung) [49]. This technique may be of value when setting PEEP and could help clinicians assess lung stresses during active breathing efforts and under conditions of high chest elastance [46, 50]. A study testing this hypothesis is currently underway (ClinicalTrials.gov NCT01681225).

Esophageal pressure measurement also enables calculation of the respiratory muscle workload and can help to detect strenuous inspiratory effort during spontaneous and assisted breathing modes. This function may be particularly important to prevent high transpulmonary pressures in the presence of high respiratory drive.

### In severe ARDS, there is no outcome advantage of using volume-controlled compared to pressure-controlled forms of ventilation

#### Rationale and literature findings

For the same tidal volume, there is no outcome advantage of using pressure-controlled versus volume-controlled ventilation in terms of the amount of stress and strain generated in the lung [51]. However, use of volume-controlled ventilation during passive inflation facilitates the measurement of respiratory mechanics and driving pressure and is recommended in the early stage. Pressure-controlled ventilation does not guarantee a fixed tidal volume, but may result in better respiratory comfort at a later stage during assisted breathing because it does not limit inspiratory flow.

### Use of high-frequency oscillatory ventilation is not recommended

#### Rationale and literature findings

Although high-frequency oscillatory ventilation (HFOV) is theoretically an attractive technique that could ensure adequate gas exchange and avoid excessive tidal stretching and atelectrauma, prospective RCTs have not shown benefit over “lung-protective” strategies implemented at conventional respiratory rates [52], and have even suggested harm [53] when used from a high pressure baseline early in the course of ARDS. Whether this technique could be used as a “rescue therapy” in very severe ARDS is unknown, but a recent meta-analysis suggests some potential advantage in these patients (P/F < ~ 70 mmHg) [54].

### Prone positioning should be used in ARDS patients with PaO_2_/FiO_2_ < 150 mmHg unless contraindicated

#### Rationale and literature findings

Prone positioning—because of its beneficial effects on oxygenation, lung recruitment and stress distribution—should be considered in the early phase of ARDS in patients with PaO2/FiO2 < 150 mmHg, and when used should be applied for 16–20 hours per day.

The physiological effects of prone positioning include redistribution of lung densities, often with recruitment of well-perfused dorsal regions. Although prone positioning increases chest wall elastance, this change is usually accompanied by improved lung recruitment, a reduction in alveolar shunt and better ventilation/perfusion ratio, with a consequent improvement in oxygenation and CO_2_ clearance, a more homogeneous distribution of ventilation and a reduction in VILI risk [55]. Not all patients with ARDS benefit from prone positioning, and lung recruitment may be central to its value. An important recent study by Guerin et al. [8] showed that prone positioning applied for at least 16 hours per day in patients with ARDS and PaO_2_/FiO_2_ < 150 mmHg significantly reduced 28-day mortality (16% vs 32%). From currently available evidence, prone positioning may be of value even if there is no improvement in gas exchange [56].

Contraindications to prone positioning include the presence of an open abdominal wound, unstable pelvic fracture, spinal lesions and instability, and brain injury without monitoring of intracranial pressure. In addition, well-trained staff are required for its safe implementation.

### In moderate/severe ARDS, neuromuscular blocking agents may be useful in the acute phase

#### Rationale and literature findings

Deep sedation alone cannot totally exclude generation of a high transpulmonary pressure and, paradoxically, can also favor certain forms of asynchrony, such as reverse triggering [50]. Neuromuscular blocking agents may be required to avoid possible dyssynchrony and the generation of excessive transpulmonary pressure by the inspiratory muscles in moderate to severe ARDS [9]. Although the benefit of this strategy may relate in part to decreased VILI as a result of lower transpulmonary pressures and reduced dyssynchrony and breath stacking [57], this speculation remains unproven. By maintaining expiratory transpulmonary pressure, neuromuscular blocking agents can prevent expiratory efforts causing derecruitment [58]. Cisatracurium can also have anti-inflammatory properties by blocking the nicotinic acetylcholine receptor [59]. However, use of neuromuscular blocking agents should be reserved for patients with the most severe ARDS, mainly in the acute phase and during the first 48 hours of mechanical ventilation. Neuromuscular blockade requires sustained deep sedation. Adverse effects of prolonged use of these drugs include myopathy, deleterious effects on the diaphragm and ICU-acquired weakness, especially in patients receiving concomitant corticosteroids [60].

### Sedation should be reduced and partial ventilator support can be used to promote respiratory muscle activity whenever gas exchange, respiratory mechanics and hemodynamic status have improved

#### Rationale and literature findings

Sedation should be titrated according to local protocols, including regular drug interruption [61]. As soon as a patient’s oxygenation improves so that the FiO_2_ and PEEP can be reduced, efforts should be taken to stop or reduce sedation and assess for weaning readiness [62]. If the patient's ventilatory drive causes high tidal volumes, an excessive respiratory rate, a profound decrease in inspiratory intrathoracic pressure or breathing discoordination, then it may be necessary to resume sedation.

Partial ventilatory support requires less sedation than fully controlled mechanical ventilation, can reduce ventilation − perfusion mismatch and can decrease the duration of ventilator support and ICU stay [63]. However, patient − ventilator synchrony is of paramount importance, and even assisted ventilation can induce VILI because of the generation of high tidal volumes and transpulmonary pressures, which if unrecognized can negatively impact patient outcome [64].

### ECMO should be considered in addition to mechanical ventilation in selected very severe cases of ARDS

#### Rationale and literature findings

ECMO can provide different degrees of CO_2_ removal and oxygenation, enabling reduction of mechanical support and VILI risk. Despite a strong physiological rationale, there is a paucity of clinical data showing that ECMO improves outcomes. Given the potentially deleterious adverse effects of ECMO, it should be reserved for the most severe cases [65] and carried out in experienced ECMO centers [10].

Some preliminary reports and a strong pathophysiological rationale suggest that ventilation with very low tidal volume (3–4 ml/kg PBW) associated with extracorporeal carbon dioxide removal (ECCO_2_R) may limit the development of VILI [66, 67]. More studies are needed before integrating this technique into a lung-protective strategy. A recent analysis suggested that it may be possible to identify those patients most likely to benefit from ECCO_2_R using physiological parameters (compliance and dead space) [68].

### Use and timing of tracheostomy should be individualized

#### Rationale and literature findings

A recent meta-analysis indicated that early tracheotomy may be associated with higher survival rates but that this may be due primarily to earlier discharge from the ICU [69]. Tracheotomy should not be used in every patient with ARDS, but should be considered when prolonged mechanical ventilation is anticipated.

### Weaning should typically be considered whenever PaO_2_/FiO_2_ > 200 mmHg with PEEP < 10 cmH_2_O, but there are exceptions

#### Rationale and literature findings

As a patient’s condition improves, the weaning process should be started based on a local protocol. The main goal of weaning is to achieve liberation from mechanical ventilation as soon as possible while limiting the risks of extubation failure.

One can consider three groups of patients with distinct characteristics and outcomes in terms of weaning [70, 71]: short, for patients in whom weaning is terminated within 24 hours after the first weaning test (up to 70% of the general ICU population); difficult, when up to 6 days are required (15% of patients); and prolonged, when 7 days or more are required (about 15% of patients). Weaning in this latter category is time-consuming and resource-consuming, associated with worse outcomes [72].

A daily SBT should be the central component of the weaning protocol, as it has consistently been shown that the duration of mechanical ventilation is significantly reduced in patients who have been assessed once daily with a period of unassisted breathing [73]. Use of a T-piece, CPAP or low levels of pressure support ventilation have been proposed for the SBT; however, clinical data are inconsistent.

For patients at high risk for extubation failure, NIV is recommended after extubation as this may significantly reduce the ICU length of stay and mortality [73]. In some specific scenarios, for patients with high risk of lung collapse (e.g., morbid obesity or in patients after cardiac surgery), direct extubation from CPAP levels ≥ 10 cmH_2_O (or PEEP ≥ 10 cmH_2_O plus low levels of pressure support) has been used with success, resulting in reduced postoperative pulmonary complications [74].

## Conclusion

Several decades of intensive research, collecting a huge amount of animal and human data, have helped modify the clinical management of patients with ARDS with a probable decrease in the overall mortality. The main goal of management should be to reduce as much as possible any potentially harmful effects of mechanical ventilation while ensuring adequate gas exchange. Targets of oxygenation, PEEP levels and use of adjuvant therapies, such as prone positioning or neuromuscular blockers, should be individualized in each patient. Use of ECMO should be considered in selected patients with reversible disease.

## Abbreviations

ARDS: Acute respiratory distress syndrome
CPAP: Continuous positive airways pressure
ECMO: Extracorporeal membrane oxygenation
FiO_2_: Inspired oxygen fraction
HFNC: High-flow nasal cannula
NIV: Noninvasive ventilation
PBW: Predicted body weight
PEEP: Positive end-expiratory pressure
Pplat: Plateau pressure
RCT: Randomized controlled trial
RSBI: Rapid shallow breathing index
VILI: Ventilator-induced lung injury
